# Photochemical Efficiency and Leaf Carbohydrates of *Theobroma cacao* L. Genotypes Under Different Light Regimes and Cultivation Systems

**DOI:** 10.3390/plants15020297

**Published:** 2026-01-19

**Authors:** Jan da Vitória, Vinicius de Souza Oliveira, Ariane Tercio Guasti, Marcos Antônio Cezario Dias, Carla da Silva Dias, Enilton Nascimento de Santana, Karin Tesch Kuhlcamp, Lúcio de Oliveira Arantes, José Altino Machado Filho, Renan Batista Queiroz, Carlos Alberto Spaggiari Souza, Edilson Romais Schmildt, Sara Dousseau-Arantes

**Affiliations:** 1 Departamento de Ciências Agrárias e Biológicas, Centro Universitário Norte do Espírito Santo, Universidade Federal do Espírito Santo, São Mateus 29932-540, Espírito Santo, Brazil; vitoriajan2@gmail.com (J.d.V.); e.romais.s@gmail.com (E.R.S.); 2 Assistência Técnica e Extensão Rural—Centro Regional de Desenvolvimento Rural—Norte, Instituto Capixaba de Pesquisa, Linhares 29901-443, Espírito Santo, Brazil; carla.dias@incaper.es.gov.br (C.d.S.D.); enilton@incaper.es.gov.br (E.N.d.S.); karin.kuhlcamp@incaper.es.gov.br (K.T.K.); lucio.arantes@incaper.es.gov.br (L.d.O.A.); altino@incaper.es.gov.br (J.A.M.F.); 3 Centro de Ciências Humanas e Naturais, Universidade Federal do Espírito Santo, Vitória 29075-910, Espírito Santo, Brazil; marcosantonio10045@gmail.com; 4 Cauliflora Consultoria e Serviços LTDA, São Mateus 29901-630, Espírito Santo, Brazil; arianetercio@hotmail.com; 5 Departamento de Agronomia, Faculdades Integradas Espírito-Santenses, FAESA, Linhares 29900-070, Espírito Santo, Brazil; renan.queiroz@f1rstagbiotech.com.br; 6 Comissão Executiva do Plano da Lavoura Cacaueira, Linhares 29900-000, Espírito Santo, Brazil; spaggiari.ceplac@gmail.com

**Keywords:** carbohydrate, chlorophyll fluorescence, photosynthetic adaptation, photosynthetic pigments

## Abstract

The cacao tree is naturally adapted to shade; however, cultivation in full-sun systems is becoming increasingly common. However, high light intensity can damage the photosynthetic apparatus, making the choice of genotype fundamental to the success of the crop. Thus, in the north of the state of Espírito Santo, municipality of Linhares, the physiological and biochemical responses of the cacao genotypes PS1319, CEPEC 2002, and PH16 were evaluated in agroforestry, cabruca, and full sun cultivation systems during the months of April to October. To this end, chlorophyll a fluorescence, photosynthetic pigments, and carbohydrates were evaluated using a completely randomized split-plot experimental design. Across agroforestry, cabruca (a traditional Brazilian shaded system), and full-sun systems, the cacao genotypes PH16, PS1319, and CEPEC 2002 did not show limitations in photosynthetic performance, as evidenced by the stable values of PI abs and PI total throughout the evaluation period. The highest quantity of photosynthetic pigments was found in the genotypes CEPEC 2002, PH16, and PS1319 in full sun cultivation, in the genotypes PH16 and PS1319 in the agroforestry system, and in the genotype CEPEC 2002 in the cabruca system. The genotypes PH16 and PS1319 obtained higher levels of glucose, sucrose, and fructose, both in shaded environments (agroforestry and cabruca systems) and in full sun. Therefore, due to their greater stability and adaptability, we recommend the PH16 and PS1319 genotypes for cultivation in agroforestry and full-sun systems, and the CEPEC 2002, PH16, and PS1319 genotypes for the cabruca cultivation system.

## 1. Introduction

The cultivation of cacao (*Theobroma cacao* L.) represents one of the most traditional and economically important agricultural activities in several tropical regions of the world. This cultivation not only drives the local economy but is also deeply intertwined with the cultures and ways of life of millions of smallholder farmers [1,2,3]. The cacao tree is native to the tropical forests of South America, more specifically the Amazon region, and its cultivation has spread to various parts of Africa, Asia, and Central America over the centuries. Currently, the world’s largest cacao producers include Ivory Coast, Ghana, Indonesia, Ecuador, Nigeria, and Brazil [2,4].

As of 2023, Brazil ranks as the world’s fifth-largest cocoa producer, with an output of 296,145 tons [5]. Brazilian cacao farming systems have significant socioeconomic implications. In many regions, cacao production is one of the few sources of income for small family farmers. Certification and fair trade initiatives have sought to value the work of these producers, ensuring better market conditions and encouraging more sustainable agricultural practices [3]. Furthermore, the chocolate industry, increasingly attentive to the traceability and sustainability of its production chains, has been pushing for positive changes in cacao production [3].

Historically, in Brazil, cacao cultivation was carried out in agroforestry systems, such as the Cabruca system, where cacao is grown under the shade of native trees, preserving part of the original biodiversity [6]. This system contributes to environmental conservation and maintains more stable microclimatic conditions, reducing the impact of pests and diseases [1,7]. However, to meet global market demand, many farmers are increasingly seeking alternative cultivation methods such as full-sun systems, which initially offer higher yields compared to shaded systems. However, without proper management, full-sun systems can lead to a higher incidence of disease and increased water and heat stress [8]. This change in the production system implies significant physiological challenges, mainly related to the absorption and use of light for photosynthesis, a central process for growth and fruit production [9].

Light is essential for plant metabolism; however, in excess it can become a stress factor, causing photoinhibition and oxidative damage to plants [10]. Excess solar radiation can compromise photosystem efficiency, impair electron transport, and negatively impact photosynthetic yield [11]. Plants grown in shaded and full sun environments develop distinct morphophysiological adaptations. Sun-exposed leaves generally exhibit greater thickness, higher stomatal density, and accumulation of carotenoid pigments, which act in protection against light stress [12,13]. In contrast, shade-exposed leaves maximize efficiency in the use of diffuse light and maintain a high concentration of chlorophyll [14]. These mechanisms are vital to ensure the integrity of the photosynthetic apparatus and the continuity of biomass production.

In cacao cultivation, when grown in shaded systems, adequate photosynthetic rates have been observed, with reduced risks of light stress in the plants [7]. However, in monocultures under full sun, despite high photosynthetic rates, which allow for high productivity, the plants, if not managed correctly with irrigation, fertilization, and pest control, can enter a state of environmental stress [15]. Furthermore, the response of cacao trees to various cultivation systems depends on the adaptability of the specific genotypes.

Studies with different cacao genotypes have been conducted aiming at selecting more productive and disease-resistant plants, as well as those adapted to different growing conditions [16]. In Brazil, the development of genotypes such as PS1319, CEPEC 2002, and PH16 has demonstrated significant improvements in productivity and bean quality [4,17]. These genotypes present desirable characteristics in relation to the fruit, such as a high proportion of pulp and high levels of ascorbic acid [18]. However, despite being notably important in the productive scenario, these genotypes still lack characterization regarding the best cultivation system to which they adapt.

It should be noted that some genotypes exhibit a greater capacity for acclimation to intense light, while others maintain superior performance under shade [2,9]. Therefore, selecting genotypes better adapted to different light conditions is strategic to ensure the productivity and resilience of cacao farming [4,9]. Thus, the objective of this study was to evaluate the physiological and biochemical responses of the cacao genotypes PS1319, CEPEC 2002, and PH16 in agroforestry, cabruca, and full-sun cultivation systems during different periods of the year.

## 2. Materials and Methods

### 2.1. Experiment Conduction

**Figure 1:**
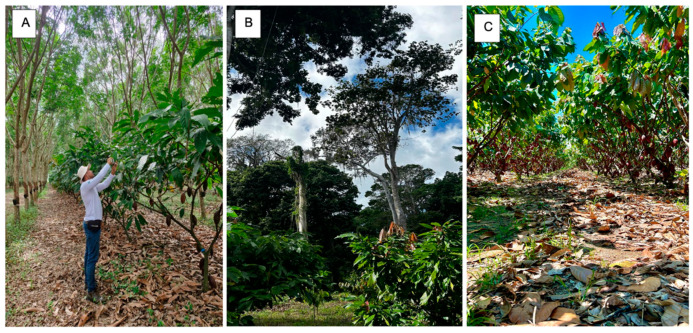
Experimental area with cacao plants grown in agroforestry (**A**), cabruca (**B**) and full sun (**C**) systems, in the municipality of Linhares, northern Espírito Santo state.

**Figure 2:**
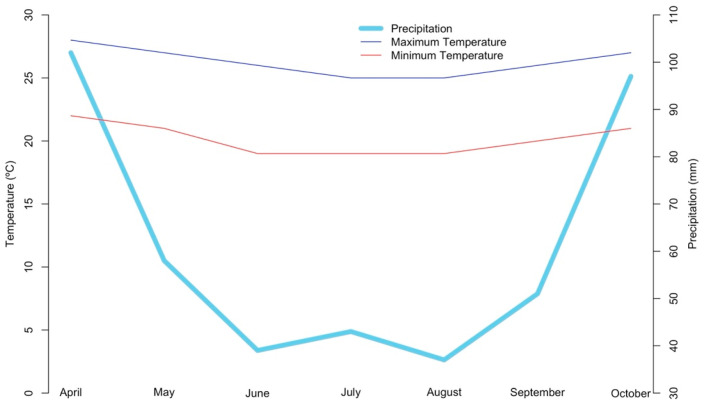
Monthly variation in maximum and minimum temperatures (°C) and precipitation (mm) from April to October, in the experimental area, in the municipality of Linhares, northern Espírito Santo state.

The study was conducted in the municipality of Linhares, in the north of the state of Espírito Santo, on three properties cultivating cacao. Each property presented a distinct cultivation system. Thus, the Sitio Nivea property (Latitude 19°16′36.74″ S, Longitude 39°58′5.97″ W) was managed in an agroforestry system (AFS) (Figure 1A), the Recanto das Oliveiras property (Latitude 19°30′5.16″ S, Longitude 40°3′31.23″ W) was managed in a cabruca system (Figure 1B), and the Chácara dos Ipês property (Latitude 19°24′1.63″ S, Longitude 40°11′41.63″ W) was managed in a full sun system (Figure 1C). The region’s climate is classified as tropical Aw with a rainy summer and a dry winter [19]. The annual temperature during the experimental period ranged between 21 °C and 26 °C. The precipitation during the experimental period ranged between 37 mm and 102 mm [20] (Figure 2).

The crops were established using seedlings obtained from certified nurseries and transplanted to the field at six months of age, with a spacing of 3 × 3 m. At the time of evaluation, the plants in the agroforestry, cabruca, and full sun systems were 5, 5, and 4 years old, respectively. In each cropping system, three cacao genotypes were evaluated: CEPEC 2002; PH16; and PS1319. Monthly evaluations were carried out throughout the year during the months of April, May, June, July, August, September, and October. Fertilization in the agroforestry system was carried out with 150 g plant^−1^ of nitrogen, phosphorus, and potassium in a 19-04-19 ratio and 300 g ha^−1^ of micronutrients via foliar application. Fertilization in the cabruca system was carried out with 400 g plant^−1^ of nitrogen, phosphorus, and potassium in a 12-11-18 ratio and 2 L ha^−1^ of micronutrients. In the full sun system, fertilization was carried out with 250 kg ha^−1^ of urea, 150 kg ha^−1^ of calcium nitrate, 400 kg ha^−1^ of KCl, 200 kg ha^−1^ of magnesium sulfate, 2 kg ha^−1^ of boron, and 40 L ha^−1^ of micronutrients. During the experimental period, no supplemental irrigation was applied.

### 2.2. Experimental Design

A completely randomized split-plot design was employed. The first factor consisted of cacao genotypes (CEPEC 2002; PH16; and PS1319). The second factor consisted of seven months of evaluation: April, May, June, July, August, September, and October. Ten plants were used per treatment, totaling 270 plants.

### 2.3. Chlorophyll a Fluorescence

During the experiment, chlorophyll a fluorescence assessments were conducted using the Pocket-PEA fluorometer (Hansatech, Norfolk, UK), following the guidelines of Strasser et al. [21]. A pair of fully expanded leaves from the middle third of the plant was used and dark-acclimated for 30 min, allowing complete oxidation of the photosystem. Then, a saturating light pulse of 3000 μmol m^−2^ s^−1^ of photons was applied for 1 s, and the parameters established by the JIP Test were obtained. Thus, the following characteristics were obtained: electron transport per reaction center (ETo RC), maximum electron retention rate per reaction center (TRo RC), absorption-based performance index (PI abs), and total photosynthetic performance index (PI total).

### 2.4. Photosynthetic Pigments

Evaluations of photosynthetic pigments were carried out monthly using the ClorofiLOG electronic chlorophyll meter (model CFL 1030, Porto Alegre, Brazil), determining the chlorophyll a and chlorophyll b indices [22]. The evaluations were performed on fully expanded leaves from the measured third of the plants.

### 2.5. Carbohydrate Extraction and Quantification

For carbohydrate evaluation, a pair of leaves from each of the three materials were collected from five plants of each genotype (CEPEC 2002, PH16, and PS1319) from each cultivation system and inactivated in a microwave at 600 watts for approximately 90 s [23]. Subsequently, the leaves were dried in a forced-air oven at 65 °C until a constant weight was reached, then ground in a ball (model TE-350, TECNAL, São Paulo, Brazil) mill for 6 min. After grinding, approximately 0.01 g of each sample was weighed using a precision analytical balance. All samples were weighed in 5 replicates. Soluble carbohydrate extraction was performed according to Pollock [24], with four extractions from the addition of 80% ethanol. The first extraction was performed by adding 1.5 mL of 80% ethanol to the tube containing the weighed samples. With the aid of a vortex mixer, the sample and ethanol were homogenized.

The samples were placed in a water bath at 80 °C for 20 min and centrifuged at 10,000 rpm for 5 min. The supernatants were reserved in 5 mL Eppendorf tubes. In the second, third, and fourth extractions, only 1 mL of 80% ethanol was added, and all the previously described procedures were repeated. The supernatants were dried in an oven for 3 days at 45 °C, and the precipitates were dried in an oven for 1 day at 45 °C.

To determine the starch content, α-amylase was diluted in MOPS buffer 120 U/mL, and amyloglucosidase was diluted in sodium acetate buffer 30 V/mL. Subsequently, 0.5 mL of α-amylase was added to the dry precipitate and incubated for 30 min at 75 °C in a water bath. Then, another 0.2 mL of α-amylase was added and incubated for another 30 min at 75 °C. After cooling to 50 °C, 0.5 mL of amyloglucosidase was added, and the samples were incubated at 50 °C in a water bath for 30 min. After this step, another 0.5 mL of amyloglucosidase was added and incubated again for 30 min at 50 °C. The samples were removed from the water bath and placed directly in the freezer. The plate reading was performed one week later, with the addition of 50 microliters of samples to an ELISA plate at 490 nm.

For the determination of glucose, fructose, and sucrose, the aqueous portion of the samples was withdrawn with an insulin syringe and filtered through a microfilter (PVDF—Filtrilo) directly into the vial, which was then frozen until the reading was taken. One week later, the reading was performed using a Shimadzu High Performance Liquid Chromatography system, with an ultrapure water mobile phase, Shim-Pack SPR-Pb column, flow rate of 0.6 mL/min, refraction detector, and a temperature of 80 °C.

### 2.6. Statistical Analysis

The data obtained were subjected to the Shapiro–Wilk normality test and analysis of variance using the F-test at a 5% probability level. When significant, the Scott–Knott clustering test was applied (*p* < 0.05). Principal Component Analysis (PCA) was also used. All analyses were performed using the R software version 4.5.2 [25], with scripts developed using the Expdes.pt package version 1.2.0 [26].

## 3. Results

### 3.1. Agroforestry System

**Figure 3:**
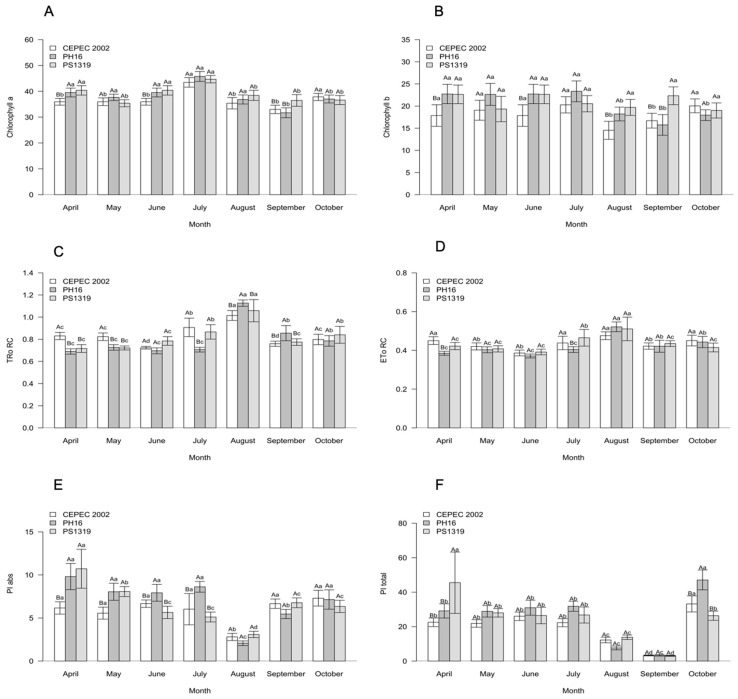
(**A**) Chlorophyll a, (**B**) chlorophyll b, (**C**) maximum electron retention rate per reaction center (TRo RC), (**D**) electron transport per reaction center (ETo RC), (**E**) absorption-based performance index (PI abs), and (**F**) total photosynthetic performance index (PI total) values in cacao plants of the genotypes CEPEC 2002, PH16, and PS1319 grown in an agroforestry system during the months of April, May, June, July, August, September, and October. Means followed by the same uppercase letter (genotypes) or lowercase letter (months) do not differ significantly according to the Scott–Knott test (*p* < 0.05). The bars indicate the standard error of the mean.

Regarding the photosynthetic characteristics of the genotypes during the evaluation months in the agroforestry system (Figure 3), it is noted that for all characteristics, there was a significant interaction between the factors. For chlorophyll a, the highest averages were observed in April in genotypes PH16 and PS1319, May in genotype PH16, June in genotypes PH16 and PS1319, July in genotypes CEPEC 2002, PH16 and PS1319, August in genotype PH16, and October in genotype CEPEC 2002. Chlorophyll b had the highest averages in April in genotypes PH16 and PS1319, May in genotypes CEPEC 2002, PH16 and PS1319, June in genotypes PH16 and PS1319, July in genotypes CEPEC 2002, PH16 and PS1319, August in genotype PS1319, September in genotype CEPEC 2002, and October in genotypes CEPEC 2002 and PS1319.

The highest average for TRo RC was observed in August in the PH16 genotype. For ETo RC, the highest averages were found in April, July, and October in the CEPEC 2002 genotypes, and in August in the CEPEC 2002, PH16, and PS1319 genotypes. The highest averages for PI abs were observed in April in the CEPEC 2002 and PS1319 genotypes, May, June, and July in the PH16 genotype, September in the CEPEC 2002 genotype, and October in the CEPEC 2002 and PH16 genotypes. For PI total, the highest averages were found in April in the PS1319 genotype and in October in the PH16 genotype.

**Figure 4:**
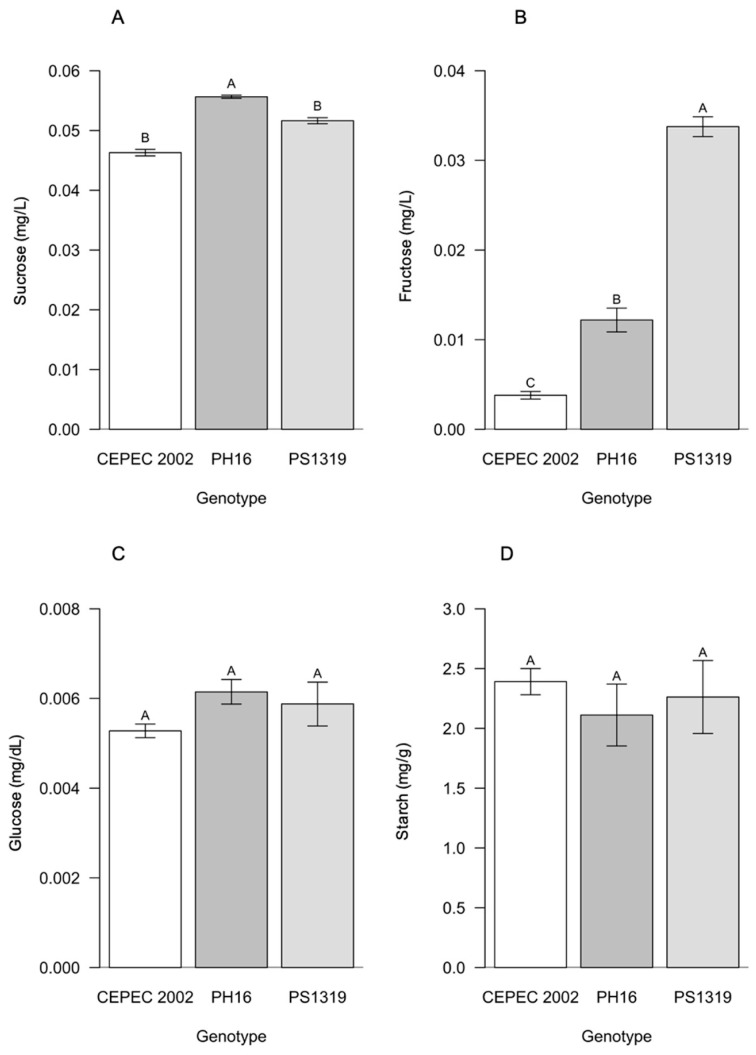
Values of (**A**) sucrose, (**B**) fructose, (**C**) glucose, and (**D**) starch in cacao plants of the genotypes CEPEC 2002, PH16, and PS1319 cultivated in an agroforestry system. Means followed by the same letter do not differ from each other by the Scott–Knott clustering test (*p* < 0.05). The bars indicate the standard error of the mean.

For the carbohydrates of the cacao genotypes in the agroforestry system (Figure 4), PH16 showed higher averages for sucrose, while the PS1319 genotype had statistically higher averages for fructose. Glucose and starch contents did not differ between the genotypes.

**Figure 5:**
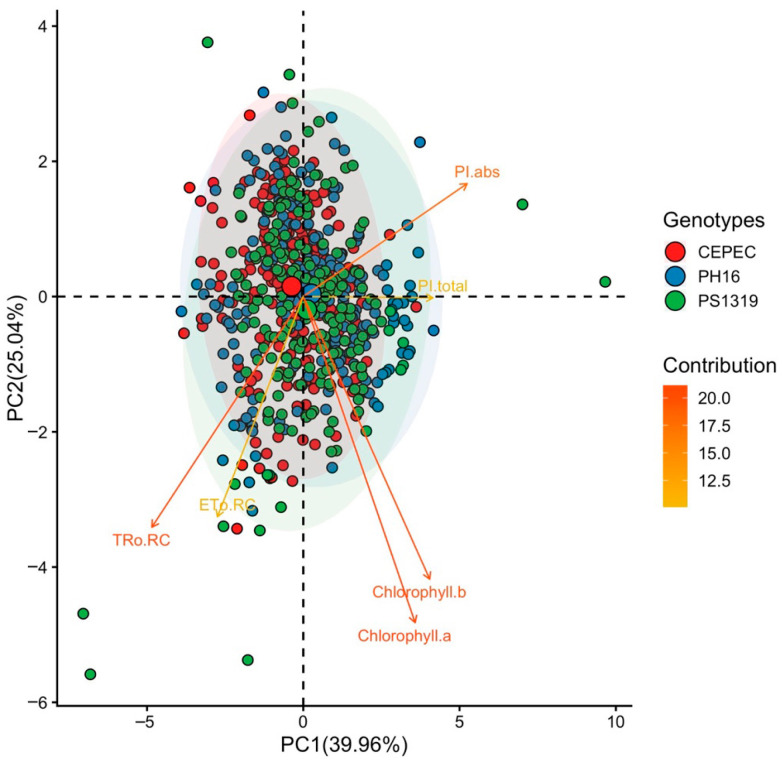
Principal component analysis (PCA) of Chlorophyll a, chlorophyll b, electron transport per reaction center (ETo RC), maximum electron retention rate per reaction center (TRo RC), absorption-based performance index (PI abs), and total photosynthetic performance index (PI total) values in cacao plants of the genotypes CEPEC 2002, PH16, and PS1319 grown in an agroforestry system.

The principal components for the agroforestry system (Figure 5) environment revealed that the first two principal components explained 39.96% (PC1) and 25.04% (PC2) of the total variance of the data, totaling 65% of the explained variability. Component 1 was responsible for the positive correlation for absolute PI, total PI, Chlorophyll a, and Chlorophyll b. On the other hand, Tro RC and ETo RC showed a negative correlation with PC1. The PH16 and PS1319 genotypes showed a positive correlation with the characteristics related to PC1.

### 3.2. Cabruca System

**Figure 6:**
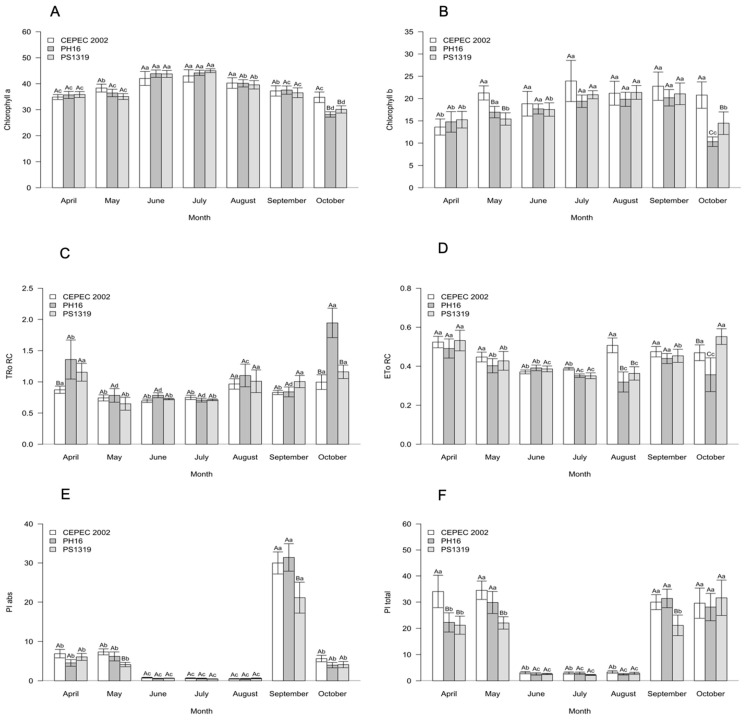
(**A**) Chlorophyll a, (**B**) chlorophyll b, (**C**) maximum electron retention rate per reaction center (TRo RC), (**D**) electron transport per reaction center (ETo RC), (**E**) absorption-based performance index (PI abs), and (**F**) total photosynthetic performance index (PI total) values in cacao plants of the genotypes CEPEC 2002, PH16, and PS1319 grown in a cabruca system during the months of April, May, June, July, August, September, and October. Means followed by the same uppercase letter (genotypes) or lowercase letter (months) do not differ significantly according to the Scott–Knott test (*p* < 0.05). The bars indicate the standard error of the mean.

In the cabruca system, all photosynthetic characteristics of the genotypes in the evaluation months showed significant interaction (Figure 6). The highest averages for chlorophyll a were found in the months of June and July in the genotypes CEPEC 2002, PH16 and PS1319 and in August in the genotype CEPEC 2002. For chlorophyll b, the statistically superior averages were observed in the months of May and October in the genotype CEPEC 2002, June in the genotypes CEPEC 2002 and PH16, and July, August and September in the genotypes CEPEC 2002, PH16 and PS1319.

The TRo RC showed higher averages in May and September in the PS1319 genotype, august in the CEPEC 2002 and PS1319 genotypes, and October in the PH16 genotype. The ETo RC had higher averages in April in the CEPEC 2002, PH16, and PS1319 genotypes, May and August in the CEPEC 2002 genotype, September in the CEPEC 2002 and PH16 genotypes, and October in the PS1319 genotype. For PI abs, the month of September in the CEPEC 2002 and PH16 genotypes was statistically superior to the others. The total Pi had higher averages in May and September in the CEPEC 2002 and PH16 genotypes and in October in the CEPEC 2002, PH16, and PS1319 genotypes.

**Figure 7:**
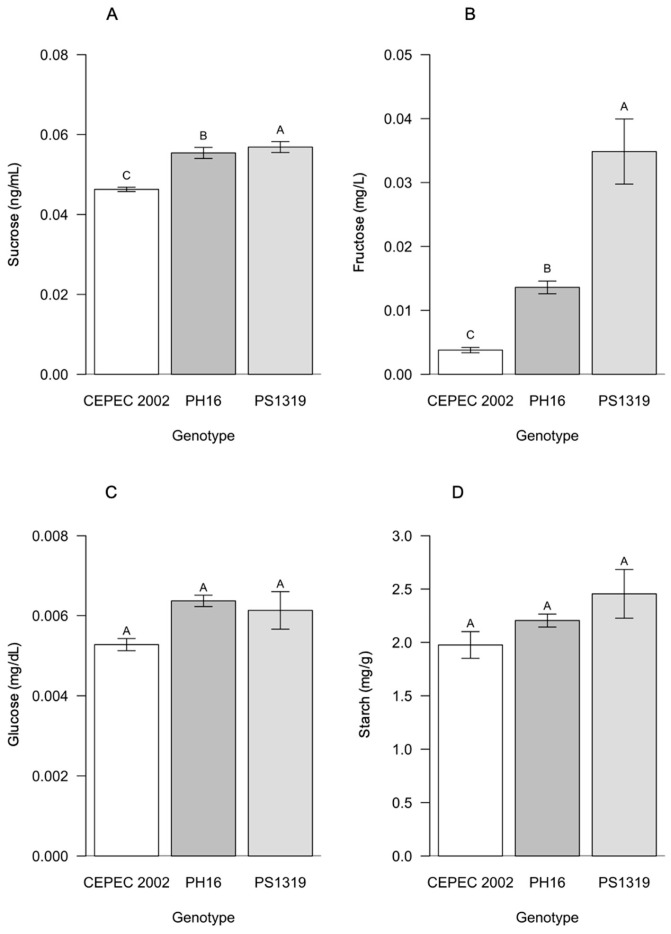
Values of (**A**) sucrose, (**B**) fructose, (**C**) glucose, and (**D**) starch in cacao plants of the genotypes CEPEC 2002, PH16, and PS1319 grown in a cabruca system. Means followed by the same letter do not differ from each other by the Scott–Knott clustering test (*p* < 0.05). The bars indicate the standard error of the mean.

Sucrose and fructose showed significant differences between genotypes, with PS1319 being superior for both characteristics compared to the other genotypes (Figure 7). For glucose and starch, no statistical differences were identified between the genotypes.

**Figure 8:**
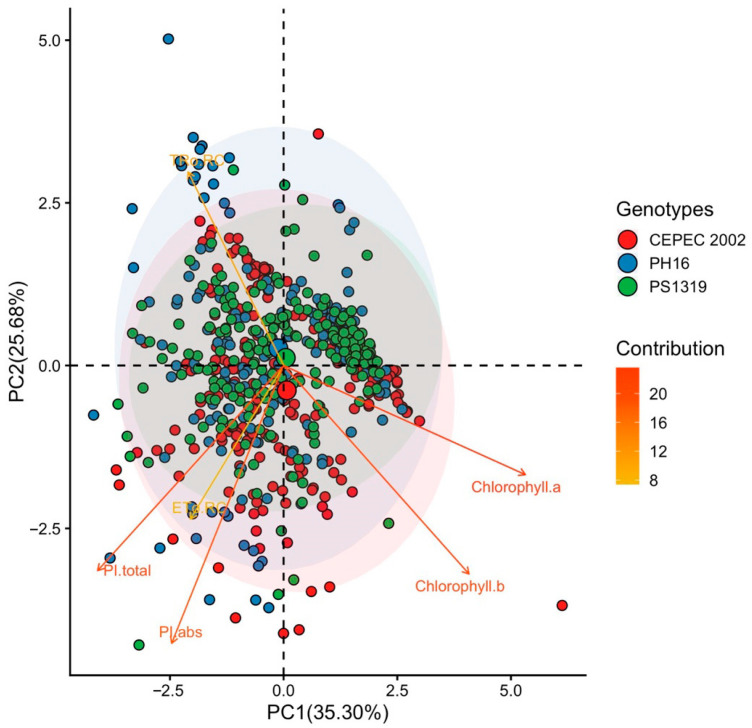
Principal component analysis (PCA) of Chlorophyll a, chlorophyll b, electron transport per reaction center (ETo RC), maximum electron retention rate per reaction center (TRo RC), absorption-based performance index (PI abs), and total photosynthetic performance index (PI total) values in cacao plants of the genotypes CEPEC 2002, PH16, and PS1319 grown in a cabruca system.

The principal component of the Cabruca system (Figure 8), the first two components explained 35.30% (PC1) and 25.68% (PC2), totaling 60.98% of the variability. The Chlorophyll a and Chlorophyll b vectors showed a strong positive association with PC1. The TRO rc vector showed a positive correlation with PC2. The CEPEC 2002 genotype showed greater proximity to the vectors linked to photosynthetic pigments.

### 3.3. Full Sun System

**Figure 9:**
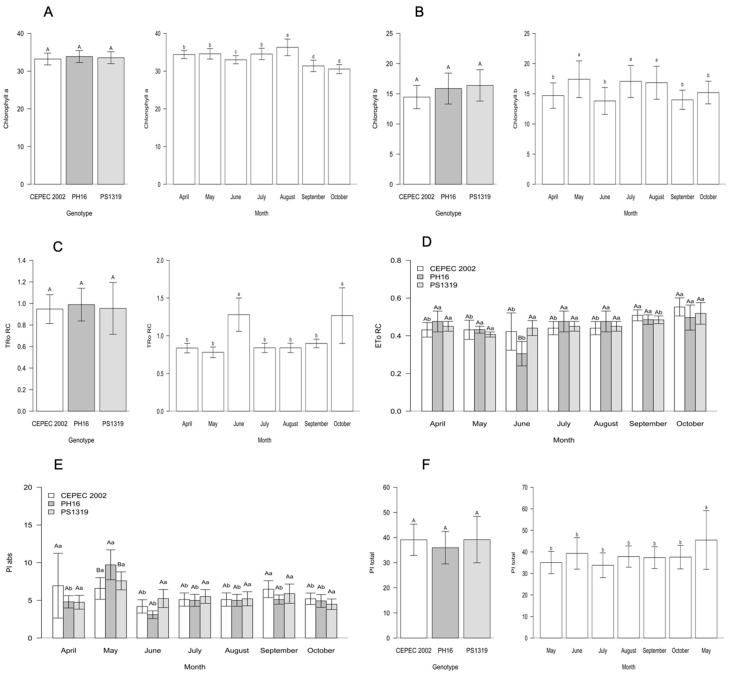
(**A**) Chlorophyll a, (**B**) chlorophyll b, (**C**) maximum electron retention rate per reaction center (TRo RC), (**D**) electron transport per reaction center (ETo RC), (**E**) absorption-based performance index (PI abs), and (**F**) total photosynthetic performance index (PI total) values in cacao plants of the genotypes CEPEC 2002, PH16, and PS1319 grown in a full sun system during the months of April, May, June, July, August, September, and October. Means followed by the same uppercase letter (genotypes) or lowercase letter (months) do not differ significantly according to the Scott–Knott test (*p* < 0.05). The bars indicate the standard error of the mean.

Regarding the genotypes and evaluation months in the full sun environment (Figure 9), a significant interaction was observed for ETo RC and PI abs. The averages were higher in genotypes PH16 and PS1319 in the months of April, May, July, and August; genotype PS1319 in June; genotypes CEPEC 2002 and PH16 in September; and genotypes CEPEC 2002, PH16, and PS1319 in October for ETo RC. PI abs had higher averages in genotypes CEPEC 2002 and PH16 in the months of April and September; genotype PH16 in May; and genotype PS1319 in the months of June, July, August, and October. For Chlorophyll a, Chlorophyll b, TRo RC, and total Pi, no significant interaction was found between the factors, nor were any statistically significant differences found for the genotypes. The highest averages were in the current month for Chlorophyll a, in May, June, and July for Chlorophyll b, in July and October for TRo RC, and in October for PI total.

**Figure 10:**
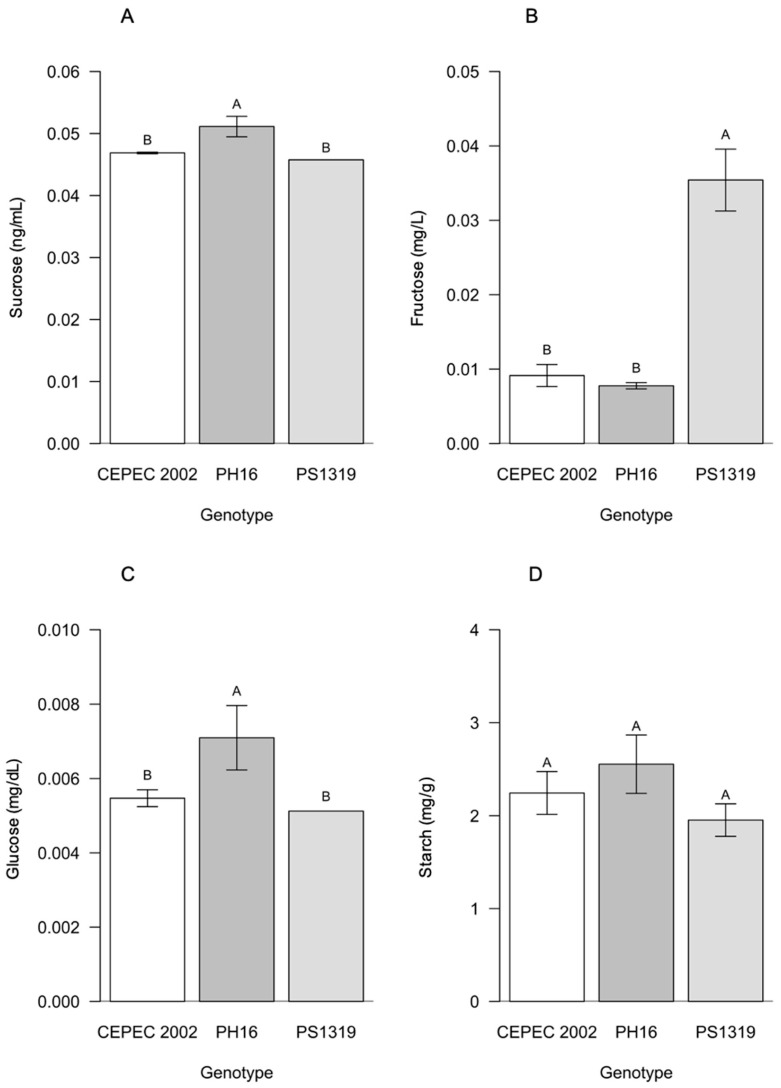
Values of (**A**) sucrose, (**B**) fructose, (**C**) glucose, and (**D**) starch in cacao plants of the genotypes CEPEC 2002, PH16, and PS1319 grown in a full sun system. Means followed by the same letter do not differ from each other by the Scott–Knott clustering test (*p* < 0.05). The bars indicate the standard error of the mean.

For carbohydrates in the full sun system (Figure 10), there were differences for sucrose and glucose, with statistically higher averages for the PH16 genotype. For fructose, the highest averages were observed in the PS1319 genotype. No significant differences were observed for starch content among the evaluated genotypes.

**Figure 11:**
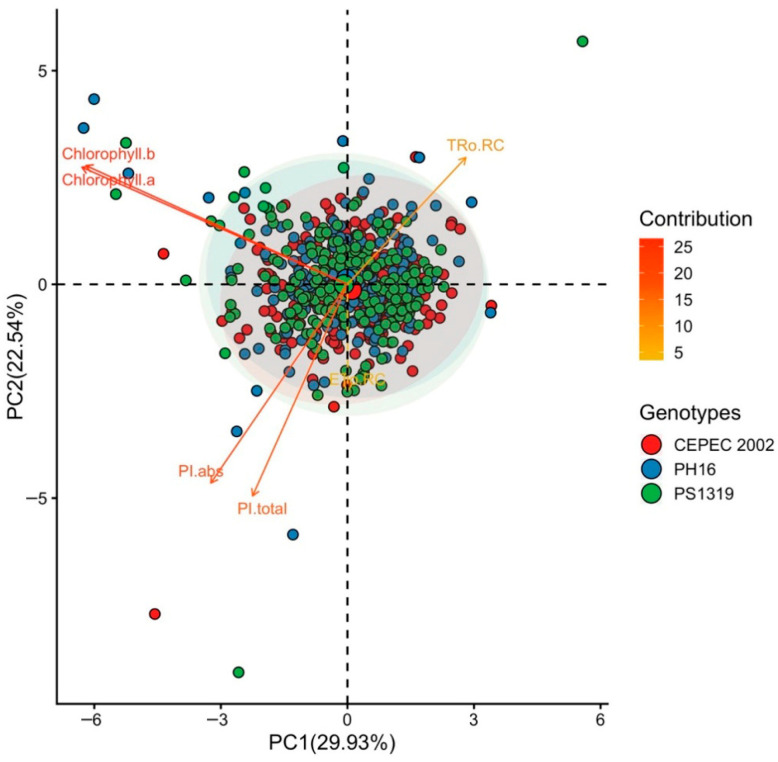
Principal component analysis (PCA) of Chlorophyll a, chlorophyll b, electron transport per reaction center (ETo RC), maximum electron retention rate per reaction center (TRo RC), absorption-based performance index (PI abs), and total photosynthetic performance index (PI total) values in cacao plants of the genotypes CEPEC 2002, PH16, and PS1319 grown in a full sun system.

The principal component in the Full Sun environment (Figure 11) explained 29.93% (PC1) and 22.54% (PC2), totaling 52.47% of the variation. In this environment, Tro RC and ETo RC showed positive results with PC1. In contrast, chlorophyll a and chlorophyll b levels shone positively with PC2. PH16 and PS1319 had a greater association with the vectors of chlorophyll levels, while CEPEC 2002 stood out for responses related to ETo RC.

## 4. Discussion

The photosynthetic apparatus, chlorophyll content, and carbohydrate content of cacao plants of the genotypes CEPEC 2002, PH16, and PS1319 were affected by the cultivation system and the months of evaluation during the year. Cacao is a typically shade-tolerant species, adapted to understory environments in humid tropical forests. However, modern agricultural practices have exposed this crop to higher light conditions, challenging the physiological plasticity of the plants [17].

Under the agroforestry system, cacao plants of genotypes PH16 and PS1319 showed higher photosynthetic performance. Genotype PH16 demonstrated greater photosynthetic capacity throughout the year, with higher averages in the months of May, June, and July for absolute PI abs. For PI total, genotypes PS1319 in April and PH16 in October were more efficient in carrying out photosynthesis in the agroforestry system. In the cabruca system, genotype CEPEC 2002 showed the highest absolute Pi value in April there was no variation for genotypes PH16 and PS1319 in the other months. In full sun, similar behavior was observed for PH16, PS1319, and CEPEC 2002, with statistically equal total Pi values among the genotypes.

According to Strasser et al. [21], PI abs and PI total are sensitive indicators of the integrity of the photosynthetic apparatus and the functional efficiency of photosystems, encompassing both light capture efficiency and energy conversion capacity. Therefore, the superior performance of PH16 and PS1319 in the agroforestry system and of CEPEC 2002 in the cabruca system may be related to better physiological adaptation to shading conditions.

Regarding TRo RC and ETo RC, although there are differences between genotypes at certain times of the year, particularly during warmer months like April and May, when the PH16 and PS1319 genotypes performed better in the full-sun system, in general, no differences were observed between the genotypes, which may indicate similar physiological behavior among them in terms of the initial dynamics of electron transport in photosystem II. According to Baker [27], the response of the electron transport chain throughout the day is directly related to the amount of photons absorbed and the plant’s ability to dissipate or use this energy efficiently. Thus, the increase in TRo RC and ETo RC in the months with higher solar radiation may indicate good efficiency in capturing and using light energy by the plants.

In cacao trees, photosynthetic capacity is crucial for vegetative growth, fruit production, and tolerance to different environmental conditions [1,9]. This process is especially sensitive to light availability, since light energy is the driving force for the conversion of carbon dioxide (CO_2_) and water into carbohydrates essential for plant metabolism and productivity. Therefore, in cacao trees, maintaining high values of PI abs and PI total is indicative of adequate photochemical efficiency and the absence of damage to photosystem II [17].

However, it should be noted that light adaptation in plants involves not only physiological changes, such as modulation of CO_2_ assimilation rate and stomatal conductance, but also morphological adjustments, such as leaf thickening and increased stomatal density, light spectral quality, water and nutrient availability, and ambient temperature [7]. These mechanisms allow plants to optimize the use of available light and reduce the risks of thermal and water stress in environments with high solar radiation.

Moderate shading has proven beneficial for cacao trees, allowing for relatively high photosynthetic rates with less risk of environmental stress [7]. On the other hand, in full sun, although it is possible to achieve high photosynthetic and productive rates, there is a greater demand for intensive agronomic management, including irrigation, fertilization, and phytosanitary control [15]. Thus, it should be considered that the ecophysiological adaptability of genotypes is a determining factor for the success of production in different environmental contexts [4]. The photosynthetic response of different genotypes in relation to the environment, as observed in our study.

Chlorophyll fluorescence has also been used as an auxiliary tool in the selection of superior genotypes. Studies have shown that cacao genotypes with greater resilience exhibit smaller variations in fluorescence parameters under stress, indicating a greater capacity for energy dissipation and protection of photosystems [4]. This can be observed in the PH16 and PS1319 genotypes in all cultivation systems studied.

For photosynthetic pigments, in the agroforestry system, the PH16 and PS1319 genotypes showed the highest values for most of the year. It is worth noting that the PS1319 genotype presented the highest chlorophyll b values in all months. In the cabruca system, the CEPEC 2002 genotype showed the highest chlorophyll a and chlorophyll b levels in august. Furthermore, the CEPEC 2002 genotype was statistically equal to the PH16 and PS1319 genotypes in June and July for chlorophyll a, and in July, August, and September for chlorophyll b. In full sun, the chlorophyll a and chlorophyll b levels were not affected during the evaluation months for the CEPEC 2002, PH16, and PS1319 genotypes.

In addition to physiological adaptations, the cacao tree also exhibits biochemical adaptations to light. The regulation of photosynthetic pigment content, especially chlorophylls a and b, is a fundamental response to the light environment [28]. According to Lichtenthaler et al. [13], higher chlorophyll levels indicate a greater capacity for light absorption and, potentially, better photosynthetic performance, provided that photo-oxidative damage does not occur. Chlorophyll a and chlorophyll b play a central role in the response to light stress and are directly related to the data obtained by fluorescence.

Plants under light stress tend to show a reduction in chlorophyll concentration [28]. These biochemical adjustments reflect evolutionary mechanisms that allow plants to cope with environments of high light variability. Thus, it is possible to observe that the genotypes CEPEC 2002, PH16, and PS1319 show adaptability to the full sun system, the genotypes PH16 and PS1319 show adaptability to the agroforestry system, and the genotype CEPEC 2002 shows adaptability to the cabruca system.

Solar radiation is a determining factor in cacao production. While cacao trees prefer shaded environments, full-sun cultivation systems have been adopted to increase productivity [17]. When grown in full sun, they frequently face challenges related to high irradiance, such as chlorophyll degradation, which can cause photoinhibition and, in extreme cases, photo-oxidative damage to plant cells [8]. Therefore, it is essential that genotypes adapted to this condition are planted in areas without shade.

Regarding the carbohydrate content in the leaves, it was observed that in the agroforestry system, the PH16 genotype stood out with the highest sucrose value, and the PS1319 genotype showed the highest fructose value. In the cabruca system, the PS1319 genotype showed the highest values for both sucrose and fructose. Sucrose is the main transport carbohydrate in higher plants, being highly sensitive to environmental conditions and the physiological status of the leaves. Therefore, these results indicate high photosynthetic capacity and efficient allocation of assimilated carbon under shading conditions for the PH16 and PS1319 genotypes, as also observed by Baligar et al. [9] and Suárez-Salazar et al. [14], demonstrating good adaptation to moderate shading of these genotypes.

In the full sun system, the PH16 genotype showed higher averages for glucose and sucrose, while the PS1319 genotype showed higher values for fructose. These results suggest that the PH16 and PS1319 genotypes exhibit greater biochemical plasticity, maintaining high carbohydrate levels even in environments with different light intensities, such as agroforestry systems, cabruca, and full sun, which may favor the transport of photoassimilates and potentially increase crop yield.

It is important to highlight that physiological adaptations to growing conditions vary among cacao genotypes. Some genotypes exhibit greater plasticity, meaning the ability to adjust their morphophysiology according to environmental conditions, while others are more sensitive to abrupt changes in light regime [4]. Knowledge of these differences is essential for producers to make appropriate genotype selections in adaptive management systems.

Practices such as the use of partial shading, whether by tree species or artificial structures, as in agroforestry and cabruca systems, can be adopted as a strategy to mitigate the impacts of thermal and light stress on cacao trees and promote a microclimate more favorable to crop development [3]. On the other hand, studies indicate that cacao genotypes grown in full sun can show significant increases in the photosynthetic rate, provided that their anatomical structures and energy dissipation mechanisms are adequately adjusted [11,17].

Multivariate analysis showed that the PH16 and PS1319 genotypes exhibited greater stability and efficiency across all systems. The CEPEC 2002 genotype showed greater efficiency, especially in shaded environments such as cabruca. Principal component analysis (PCA) identified patterns of multivariate variation in the photochemical parameters of cacao clones cultivated in different environments. The model robustly explained the variability of the data, with the first two principal components (PC1 and PC2) representing more than 52% of the total variance in all systems analyzed.

These results reinforce the importance of genotype–environment interaction in the photosynthetic response of cacao trees. The combination of multivariate analysis with detailed physiological parameters allowed for a clear discrimination of genotypes with greater adaptability to different environments.

In shaded environments (agroforestry system and Cabruca), the variables related to chlorophyll content clustered strongly, positioning themselves in the same quadrant in the biplots, which demonstrates the consistency of these variables as markers of physiological performance in different shaded environments. In agroforestry and full sun environments, the PH16 and PS1319 genotypes show a greater relationship with chlorophyll content. These results suggest that these clones exhibit greater physiological plasticity, being able to modulate the photosynthetic apparatus according to light availability, which is fundamental for adaptation to different light regimes [9,29].

It is worth highlighting that, in climate change scenarios, understanding the anatomical and morphological responses of the cacao tree to solar radiation becomes even more crucial. Increased temperatures, coupled with intensified drought periods and higher radiation incidence, may require plants with greater structural adaptation capacity [8].

The ideal temperature for cacao tree development is between 21 °C and 32 °C. Temperatures below or above this range can vary depending on photosynthesis, abundance, and pod filling [15]. In regions with rising average temperatures, as a result of global climate change, there is a higher occurrence of flower and fruit abortion, directly impacting production [3].

In climate change scenarios, understanding the anatomical and morphological responses of the cacao tree to solar radiation becomes even more crucial. Increased temperatures, coupled with intensified drought periods and higher radiation incidence, may require plants with greater structural adaptation capacity [8].

Studies indicate that changes in forecasting patterns, rising temperatures, and the increased frequency of extreme weather events can severely impact the productivity and forecasts of traditionally cultivated areas [15]. Adapting to climate change involves developing more resilient cultivars, improving agronomic management, and adopting diversified and sustainable cropping systems [7].

Climate change poses an additional challenge to global cacao farming. Projections indicate that many currently producing areas may become unsuitable for cultivation due to rising temperatures and reduced rainfall [8]. Therefore, adapting agriculture through improved agroforestry systems and developing policies to support farmers is fundamental to ensuring the continuity of production [3].

Environmental variations also have implications for the quality of cacao beans. Factors such as light, temperature, and water availability influence the concentration of bioactive compounds, such as theobromine, caffeine, polyphenols, and flavonoids, which are crucial for the flavor, aroma, and nutritional value of chocolate [1].

In studies conducted in regions of Mexico, cacao clones grown under different microclimate conditions showed variations not only in productivity but also in bean quality, highlighting the importance of the environment on the phenotypic expression of the plant [7]. Therefore, the ecophysiological plasticity of genotypes is a determining factor for adaptation and production success in different environmental contexts [4].

Thus, the influence of environmental conditions on cacao production is multifactorial and complex, requiring an integrated approach that combines genetics, agronomic management, environmental conservation, and public policies. Understanding these interactions is key to ensuring the resilience and sustainability of cacao farming in the present and the future.

Therefore, photosynthesis and light adaptation in cacao plants constitute a fundamental field of study for the development of more productive and resilient cultivation systems. Understanding the physiological, morphological, and biochemical responses of the cacao tree to light allows producers and researchers to make more informed decisions about crop management under different environmental conditions. Thus, ensuring the health and productivity of the cacao tree requires an integrated approach that takes into account both the plant’s physiological requirements and contemporary ecological and climatic challenges.

The ecophysiological plasticity of the cacao tree is also manifested in the ability of different genotypes to respond to contrasting light environments. Studies have shown that some genotypes exhibit better photosynthetic performance and higher productivity under high light levels, while others maintain better stability under shading [4]. Therefore, the selection of adapted genotypes and proper light management are essential to ensure photosynthetic efficiency and the sustainability of cacao production in different environments [3].

Thus, in summary, as demonstrated in our studies, the cacao genotypes PH16 and PS1319 showed great adaptability and stability in both moderately shaded environments, such as in agroforestry systems, and in open soil, and can be indicated for different light conditions. Furthermore, the genotypes CEPEC 2002, PH16, and PS1319 proved suitable for cultivation in environments with greater shade, such as the cabruca system. It is worth highlighting that choosing a genotype adapted to growing conditions is only one strategy; other factors such as proper management of nutrition, water resources, and phytosanitary conditions are essential to guarantee the success of the crop.

## 5. Conclusions

The cacao genotypes PH16, PS1319, and CEPEC 2002 did not show limitations in photosynthetic performance in agroforestry, cabruca, and full-sun cultivation systems, as evidenced by the stable values of PI abs and PI total throughout the experimental period.

Regarding photosynthetic pigments, the genotypes CEPEC 2002, PH16, and PS1319 show adaptability to the full sun system, the genotypes PH16 and PS1319 show adaptability to the agroforestry system, and the genotype CEPEC 2002 shows adaptability to the cabruca system.

The PH16 and PS1319 genotypes stood out with higher levels of glucose, sucrose, and fructose found in the leaves, both in shaded environments (agroforestry and cabruca systems) and in full sun. This indicates high photosynthetic capacity and efficient allocation of assimilated carbohydrates in environments with different light intensities, potentially suggesting higher yields.

Therefore, we recommend the PH16 and PS1319 genotypes for cultivation in agroforestry and full sun systems, and the CEPEC 2002, PH16, and PS1319 genotypes for the cabruca cultivation system. However, the choice of genotype alone is not a guarantee of crop success; cultural practices such as nutrition, water, and pest and disease management must be adjusted according to each cropping system, allowing for greater sustainability of the plantings.

## Data Availability

The original contributions presented in this study are included in the article. Further inquiries can be directed to the corresponding authors.

## Abbreviations

The following abbreviations are used in this manuscript:

ETo RC	electron transport per reaction center
TRo RC	maximum electron retention rate per reaction center
PI abs	absorption-based performance index
PI total	total photosynthetic performance index
